# ISMRM Clinical Focus Meeting 2023: “Imaging the Fire in the Brain”

**DOI:** 10.1002/jmri.29587

**Published:** 2024-08-28

**Authors:** Nivedita Agarwal, Audrey Fan, Xiaoqi Huang, Seena Dehkharghani, Anja van der Kolk

**Affiliations:** ^1^ Diagnostic Imaging and Neuroradiology Unit IRCCS Scientific Institute E. Medea Bosisio Parini Lecco Italy; ^2^ Department of Neurology University of California Davis Health Sacramento California USA; ^3^ Department of Biomedical Engineering University of California Davis Davis California USA; ^4^ Department of Radiology and Huaxi MR Research Center (HMRRC), Functional and Molecular Imaging Key Laboratory of Sichuan Province, West China Hospital Sichuan University Chengdu China; ^5^ Department of Radiology Albert Einstein College of Medicine‐Montefiore Health New York New York USA; ^6^ Department of Medical Imaging Radboudumc Nijmegen The Netherlands

**Keywords:** neuroinflammation, MRI, PET, autoimmune disorders, brain tumors and patient oriented research

## Abstract

Set during the Annual Meeting of the International Society for Magnetic Resonance in Medicine (ISMRM), the “Clinical Focus Meeting” (CFM) aims to bridge the gap between innovative magnetic resonance imaging (MRI) scientific research and daily patient care. This initiative is dedicated to maximizing the impact of MRI technology on healthcare outcomes for patients. At the 2023 Annual Meeting, clinicians and scientists from across the globe were invited to discuss neuroinflammation from various angles (entitled “Imaging the Fire in the Brain”). Topics ranged from fundamental mechanisms and biomarkers of neuroinflammation to the role of different contrast mechanisms, including both proton and non‐proton techniques, in brain tumors, autoimmune disorders, and pediatric neuroinflammatory diseases. Discussions also delved into how systemic inflammation can trigger neuroinflammation and the role of the gut‐brain axis in causing brain inflammation. Neuroinflammation arises from various external and internal factors and serves as a vital mechanism to mitigate tissue damage and provide neuroprotection. Nonetheless, excessive neuroinflammatory responses can lead to significant tissue injury and subsequent neurological impairments. Prolonged neuroinflammation can result in cellular apoptosis and neurodegeneration, posing severe consequences. MRI can be used to visualize these consequences, by detecting blood–brain barrier damage, characterizing brain lesions, quantifying edema, and identifying specific metabolites. It also facilitates monitoring of chronic changes in both the brain and spinal cord over time, potentially leading to better patient outcomes. This paper represents a summary of the 2023 CFM, and is intended to guide the enthusiastic MR user to several key and novel sequences that MRI offers to image pathophysiologic processes underlying acute and chronic neuroinflammation.

**Evidence Level:**

5

**Technical Efficacy:**

Stage 3

The term “inflammation” originates from the Latin “*inflammare*,” which literally translates as “*to set on fire*.” It was initially coined by the Roman encyclopedist Aulus Cornelius Celsus. Inflammation of tissues is typically characterized by a set of signs and symptoms including *calor* (heat), *rubor* (redness), *tumor* (swelling), and *dolor* (pain). While these are easily identifiable when inflammation affects the skin and other body organs, tissues within the central nervous system (CNS) undergoing inflammation respond differently and are hidden from direct inspection, making clinical diagnosis difficult.

Neuroinflammation encompasses a distinct set of, responses within the CNS. Its primary benefit is to minimize tissue damage, facilitate injury repair, and offer neuroprotection.^1^ It can be triggered by infection, external injury, or autoimmune reactions, and can manifest as acute or chronic. It encompasses intricate molecular and cellular pathways, including neuronal activation, release of inflammatory mediators, and immune cell activation and infiltration, potentially leading to neuronal damage and dysfunction. Interactions with neurogenesis and synaptic plasticity are also notable.^2^ Biomarkers of neuroinflammation detected in blood, cerebrospinal fluid or other bodily fluids are critical for studying and understanding this process, aiding in disease diagnosis, monitoring, and treatment. Many neurologic disorders feature at least some degree of associated inflammation, driven by disturbances in CNS homeostasis and involving innate and adaptive immune responses aimed at limiting disease progression and promoting repair. However, the intensity and duration of neuroinflammatory reactions can lead to adverse outcomes such as cellular apoptosis, edema, and dysfunction of neuronal‐glial interactions^3^, ^4^ (Fig. 1). Neuroinflammatory processes prolonged over years may culminate in neurodegeneration, diminished cognitive capacity, and the onset of psychiatric illness.^5^

**FIGURE 1:**
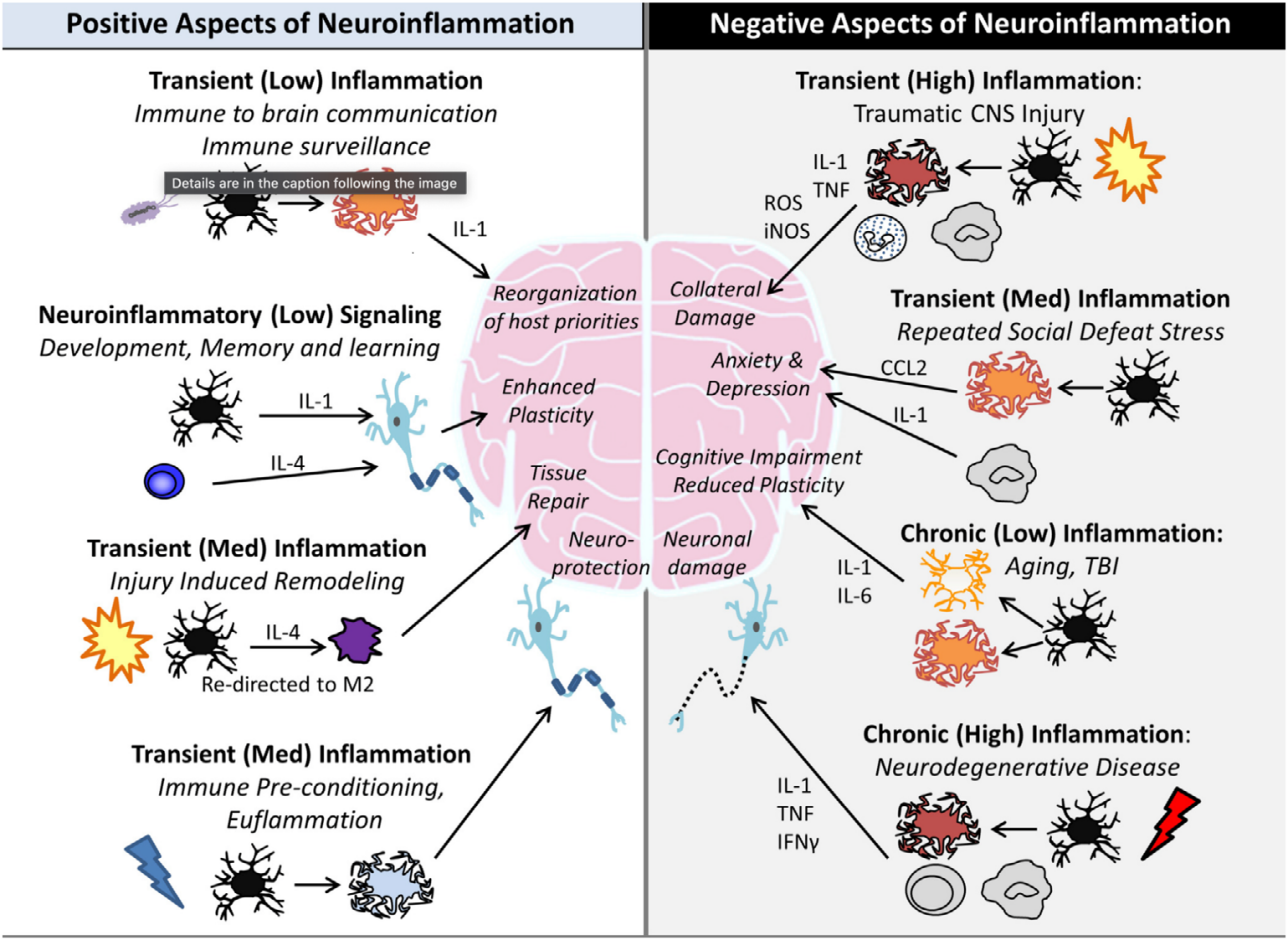
Positive and negative aspects of neuroinflammation. The intensity and duration of inflammation account for much of whether immune signals are supportive or destructive to the central nervous system.^3^

During the 2023 Annual Meeting of the International Society of Magnetic Resonance in Medicine (ISMRM) in Toronto, a three‐day Clinical Focus Meeting (CFM) entitled “Imaging the Fire in the Brain” was convened, which brought together experts in the field to explore various aspects of neuroinflammation, its significance in neurological conditions, emerging magnetic resonance‐based imaging methods, and potential future directions in neuroimaging. The presentations were delivered by a diverse group of professionals, predominantly clinicians, who shared their insights into the utilization of MRI across a broad spectrum of neurological disorders. This paper is intended to guide the enthusiastic MR user through these topics as introduced during the CFM. After discussing key mechanisms and both established and emerging MRI techniques for imaging neuroinflammation, we provide several clinical examples of how clinicians could employ these techniques in practice. As this is essentially a meeting summary, not all techniques and neurological diseases will be discussed, and the interested reader is referred to several excellent resources offering a more general overview of neuroinflammation.^6^, ^7^

## MRI‐Based Contrast Mechanisms for Imaging Neuroinflammation

**FIGURE 2:**
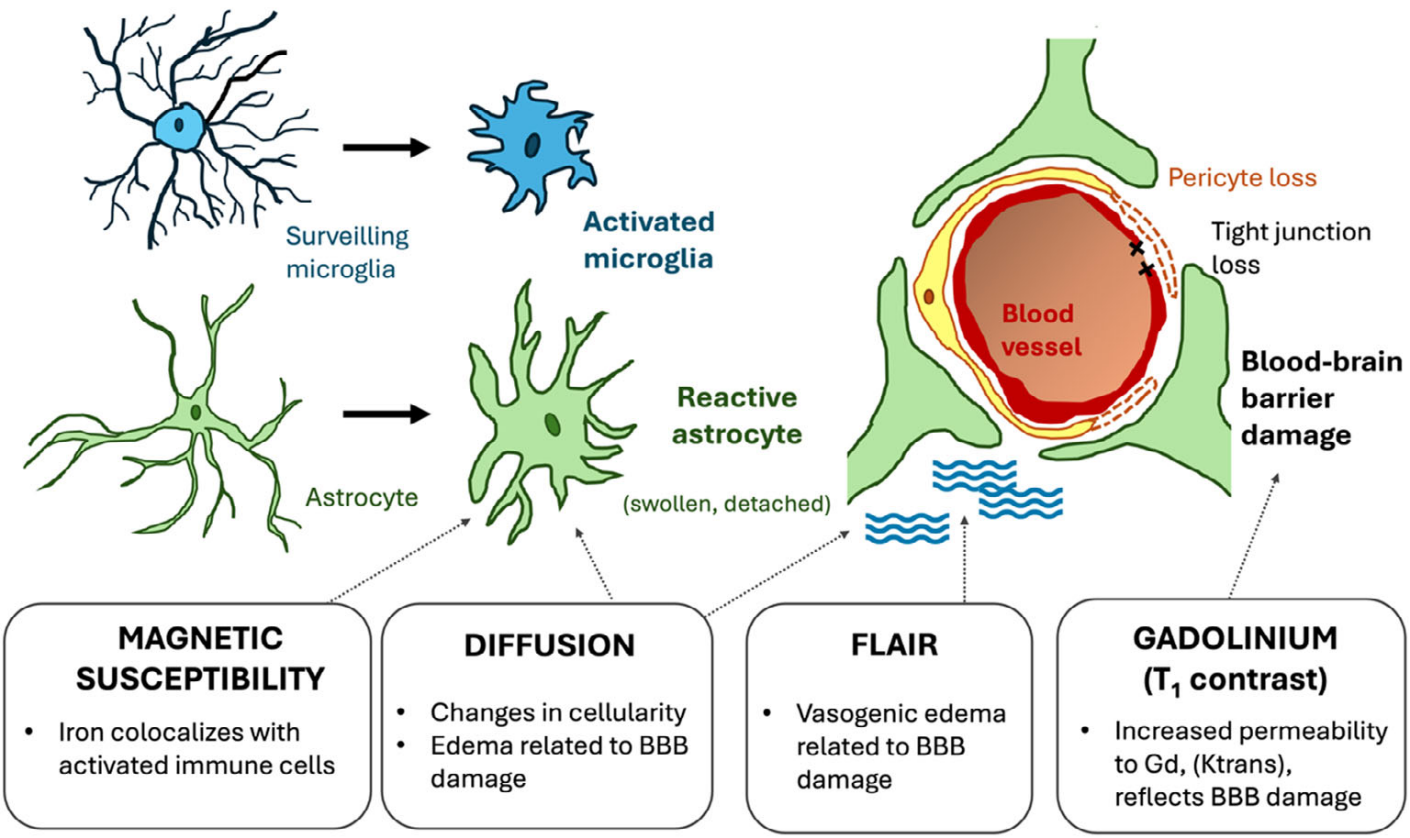
Contrast MRI contrast mechanisms of neuroinflammation include magnetic susceptibility (gradient echo or quantitative susceptibility mapping), diffusion MRI, dynamic susceptibility contrast and FLAIR MRI and are linked to cellular changes in inflammatory cells and the neurovascular unit. This figure has been modified from Palpagama et al.^8^

The detection of neuroinflammation in routine neuroimaging is generally based on two main features: 1) edema—by means of T_2_‐weighted (FLuid‐Attenuated Inversion Recovery [FLAIR]) imaging and 2) blood–brain barrier (BBB) integrity—most commonly using tissue enhancement following administration of exogenous paramagnetic contrast agents with T_1_ weighting (Fig. 2).^8^ While useful in standard clinical practice, these features lack specificity for inflammation, making the diagnosis of inflammatory lesions in the CNS challenging. In this CFM educational program, iron and diffusion tensor imaging (DTI) were highlighted as other key contrast mechanisms, since iron can serve as a proxy for neuroinflammation and has paramagnetic properties on multiple MRI sequences, and DTI can visualize microstructural changes associated with inflammation. Some of the associated new imaging biomarkers, such as the paramagnetic rim sign observed in multiple sclerosis, enhance imaging specificity but are applicable only to certain neuroinflammatory conditions.^9^ Nevertheless, these developments hold promise for clinical translation, potentially improving the diagnosis and characterization of neuroinflammatory diseases.

### Exogenous Iron

Iron can be used either as exogenous or endogenous contrast agent. As an exogenous contrast agent, iron is incorporated in ultra‐small superparamagnetic iron oxide nanoparticles (USPIOs), such as magnetite or other iron oxides, and can be used to tag cells.^10^ After intravenous administration of USPIOs such as ferumoxytol into the body, these particles are taken up by phagocytes in the liver, spleen and bone marrow, but can also be internalized by reactive astrocytes, microglia, and dendritic cells in the CNS.^11^, ^12^ This uptake determines a strong signal attenuation in T_2_*‐weighted images, increasing sensitivity to the presence of a local immune response. However, remaining technical challenges include low specificity because extracellular iron is already present in some areas of the brain, and a lack of ability to quantify cell numbers (which vary with cell size, imaging parameters, and relaxation regime). The outlook for neuroinflammation imaging with exogenous iron thus includes development of smaller and targeted superparamagnetic iron oxide nanoparticles to reveal specific inflammatory processes (eg, vascular adhesion in blood vessel inflammation) and to enable tracking for drug delivery. Newer contrast agents can be further combined with novel molecular imaging approaches such as *magnetic particle imaging* in order to push the limit of cellular detection and further improve sensitivity and specificity.^13^

Exogenous USPIO contrast on T_2_*‐weighted MRI can also be difficult to interpret in the presence of BBB breakdown that frequently occurs with neuroinflammation. Because USPIOs can accumulate in tissues with leaky BBBs and change T_2_*‐weighted MRI signal, iron‐sensitive scans may be complemented by dynamic contrast enhanced (DCE) MRI using more conventional gadolinium chelates with the potential benefits of more direct assessment of BBB integrity.^14^ DCE MRI leverages dynamic delivery of gadolinium contrast (eg, 25 mL at 3 mL/s). MRI acquisitions include an initial T_1_‐map, followed by dynamic T_1_‐weighted scans through inversion recovery, variable flip angle, or dual‐time saturation recovery pulse sequences. The dynamic signal is then fit to a two‐compartment Tofts model that captures Gd contrast passage from blood into tissue, resulting in an estimate of K_trans_ (transfer rate) as a marker of BBB permeability.^14^ Although DCE MRI is well‐known to measure acute BBB changes in stroke and tumor, and even more subtle BBB changes in neurodegeneration,^15^ the CFM course pointed to wide discrepancies in reported K_trans_ values due to variable acquisition and modeling strategies.^16^ This observation points to the need for harmonization of DCE protocols for use for neurological conditions to assess BBB changes concomitant with neuroinflammation.

### Endogenous Iron

**FIGURE 3:**
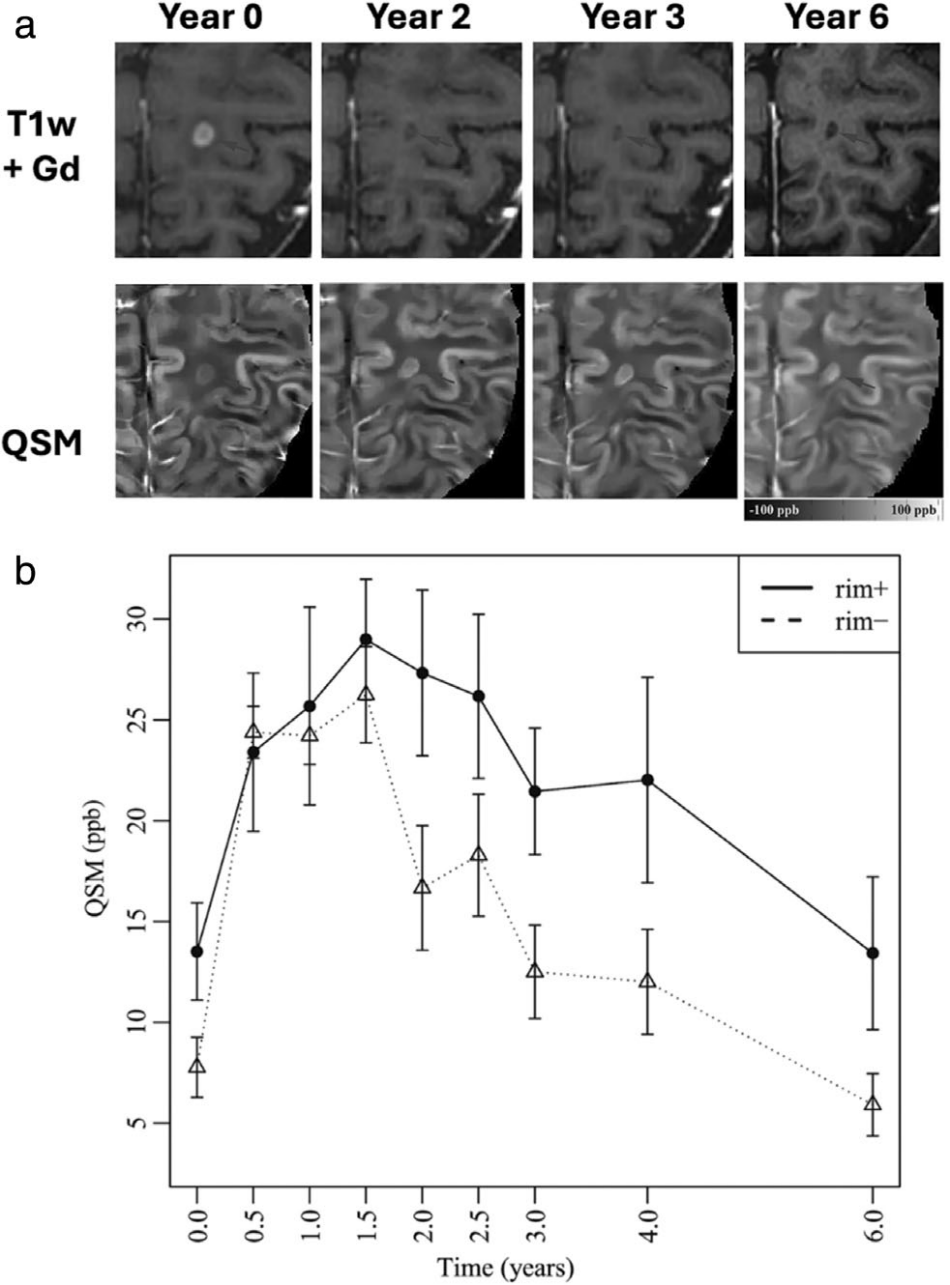
Longitudinal QSM and T2‐weighted FLAIR images of a new Gd‐enhancing multiple sclerosis (MS) lesion with a QSM rim appearance (rim‐positive). (**a**) The lesion was slightly hyperintense on QSM (black arrow) at the time of Gd‐enhancement, became more hyperintense at 3 years, and remained hyperintense at 6 years. (**b**) Measurements from QSM in a 32 new Gd lesions from 19 patients with relapsing–remitting MS. Rim‐positive lesions demonstrate higher peak QSM value, indicative of iron‐laden inflammatory cells, and significantly slower decay rate compared to rim‐negative lesions This figure has been adapted from Zhang et al.^24^

Separately, iron also serves as a source of endogenous contrast for neuroinflammation because brain microglia are iron‐rich.^17^, ^18^ Consequently, a T_2_*‐sensitive multi‐echo gradient echo MRI scan can reveal paramagnetic changes as T_2_*‐signal reduction colocalized to increased presence of microglia even without exogenous contrast administration. At the same time, these gradient echo scans provide phase (or field map) information, which can be used to reconstruct a quantitative susceptibility map (QSM) of tissue magnetic susceptibility through a mathematical inversion of the MRI magnetic dipole.^19^ Having QSM maps from the same scan can help disentangle the presence of other molecules (eg, myelin, calcium deposits) that have opposite diamagnetic effects to iron, but are hard to separate on T_2_*‐weighted images.^20^ In studies of multiple sclerosis, the presence of QSM‐positive paramagnetic rims around white matter lesions has been co‐localized with CD68+ microglia and macrophages on histology. These rims also show increased binding of the PET ligand ^11^C‐PK11195 to translocator protein (TSPO), which is expressed on the membranes of activated microglia and macrophages.^21^ QSM is an ideal noninvasive method to assess paramagnetic rim lesions while avoiding dipole spreading effects on original phase scans that can create false positive detections. The paramagnetic rim lesions on MRI have been shown to be specific to MS vs. other neurodegenerative disorders, can be monitored longitudinally (Fig. 3), and represent a clear therapeutic target for intervention.^9^ Patients with at least one positive‐rim lesion on QSM maps show cognitive reductions in multiple domains and reduced cortical thickness.^22^

In neurodegenerative disorders, especially of the white matter, neuroinflammatory processes may co‐occur with microstructural changes such as demyelination and remyelination that are hard to disentangle. QSM methods that model and create separate maps of paramagnetic and diamagnetic susceptibility contributions can thus enable more accurate quantification of neuroinflammatory processes. If paired with suitable automated segmentation methods, QSM and T_2_*‐weighted MRI methods can lead to standardization in the MS community on the definition of a clinically relevant, positive‐rim lesion.^23^, ^24^ These endogenous iron imaging methods can then be extended to detect subtle and widespread chronic and inflammatory signatures longitudinally in neurodegenerative diseases.

### Microstructural Changes Using Diffusion Imaging

Microstructural changes associated with neuroinflammation have been studied with diffusion imaging. Using diffusion tensor or diffusion kurtosis signal models, the extracted parameters of water diffusivity and kurtosis (i.e., non‐Gaussian skewness of water diffusion) are sensitive to different aspects of neuroinflammation.^25^, ^26^ For instance, in its activated state, microglia have fewer extended processes of the cell, which leads to a reduction in mean diffusivity (MD). The presence of microglia and macrophages also leads to increase in mean kurtosis (MK) due to increased cellular heterogeneity.^27^, ^28^ However, the effect size of these changes may be reduced by the presence of vasogenic edema and demyelination in the same tissues during disease, both of which increase MD.^29^ Cytotoxic edema has the same effect (reduced MD, increased MK) as microgliosis and therefore can be confused with neuroinflammation. Overall, the sensitivity of diffusion measures (due to abundance of water in brain tissues) is balanced by the lack of measures specific to neuroinflammation. This technical limitation is being addressed with current diffusion modeling work that considers different biophysical compartments (cylindrical vs. spherical compartments, for instance) to isolate characteristics of glia vs. neurons.^30^, ^31^ Diffusion measures can also be combined with molecular specificity obtained with spectroscopy, one among several innovative molecular imaging strategies with MRI that are discussed in the next section.

## Novel Contrast Mechanisms for Imaging Neuroinflammation

Beyond the more established approaches introduced above, there exist exciting opportunities to expand the currently available techniques for clinical and translational study of neuroinflammation. A key goal across several talks on advanced contrast mechanisms during the CFM was to improve the molecular specificity of MRI techniques.

### MR Spectroscopy

Proton and non‐proton spectroscopy (MRS) have been discussed extensively for their ability to identify altered or aberrant metabolite concentrations that portend inflammatory conditions in the brain. Standard implementations of proton spectroscopy attainable on most clinical systems have been explored for their detection and quantification of choline (Cho), lactate, or myoinositol (Ins) levels as potential indicators of accelerated cellular turnover, altered oxidative metabolism, or myeloid and glial cell activation, respectively. The metabolites of interest are small molecules that are intermediate or end‐products of cellular metabolism that occur in low concentrations preferentially in certain brain cell populations. For instance, neuroinflammation is associated with increased total choline (tCho) and Ins, which reflect membrane turnover and osmotic changes, and with elevated creatine levels, which reflect more availability and transfer of energy in glial cells.

### Diffusion MRS

Diffusion magnetic resonance spectroscopy leverages a combination of diffusion encoding and spectroscopic readout in one novel acquisition to provide direct access to metabolites in the intracellular space of inflammatory cells. By tailoring diffusion gradients to capture short (0.1–10 msec), intermediate (13–250 msec), and long (500 msec–10 s) diffusion times, the resulting metabolite signal can be modeled to separately reflect the cytosol viscosity, fiber and soma radius, and the longer cell extensions/cell complexity of the glia, respectively.^32^ During neuroinflammation, the soma diameter, diameter of glial processes, and length of glial processes will increase while overall cell complexity decreases.^32^ Because the specific diffusion of tCho and Ins compounds in these small compartments will reflect how these compartments change in size during neuroinflammation, diffusion MRS can provide targeted morphological and metabolic information about activated glia and reactive astrocytes.^33^

The CFM highlighted several advanced applications of diffusion MRS in human studies, particularly the accessibility of measuring apparent diffusion coefficient (ADC) of tCho even at standard field strengths. For instance, glial‐enriched tCho signals from diffusion MRS were elevated in early stages of schizophrenia in patients with first episode psychosis, whereas no significant disease‐related changes were observed in standard water‐based measures of fractional anisotropy, mean diffusivity, or T2 relaxation.^34^ Elevated ADC of tCho was also observed in patients with ischemic stroke 1 month post‐stroke, indicating glial morphometric changes associated with active inflammation at that time‐point.^35^ In the same study, high diffusivity of total creatine up to 3 months after the ischemic event was observed, indicating continued astrogliosis in chronic stages after stroke recovery. Diffusion MRS also detected changes in ADC of tCho during systemic inflammation induced by lipopolysaccharide (LPS) injection in human volunteers, specifically in the thalamus, which is known to have high basal microglia density.^36^ As with other spectroscopic applications, considerable attention is required in the processes of volume localization in 2D or 3D modes, selecting echo times to capture fast‐decaying metabolites, managing signal‐to‐noise ratio limitations and requisite signal averaging when using small voxel sizes, maintaining robust shim conditions, and addressing variability in excitation profiles and chemical shift displacement at higher fields. Both stimulated and spin echo readouts have been used for diffusion MRS, and detailed consideration of cross‐term effects of other (non‐diffusion) gradients in the signal model is critical for successful quantification of metabolite diffusion properties.

### Chemical Exchange Saturation & Magnetization Transfer

Magnetization transfer (MT) and chemical exchange saturation transfer (CEST) MRI are related, “label‐free” approaches that use similar acquisitions to image endogenous biochemicals with relevance to neuroinflammation. MT and CEST methods label protons on the target metabolites using off‐resonance saturation pulses at specific corresponding frequencies, and this proton signal then transfers to water molecules to effectively amplify the signal over 100‐fold compared to MR spectroscopy. Although the readout frequency for MT and CEST is also at the water frequency (such as a basic echo planar imaging), the signal amplification in these methods overcomes some challenges of low SNR in spectroscopic imaging. Outcome measures of these methods include magnetization transfer ratio (MTR) based on a two‐pool exchange model or the asymmetry of MTR on the spectral line shape (or Z‐spectra).^37^, ^38^ These measures are semi‐quantitative in most implementations due to hardware and acquisition considerations, thus limiting generalizability and use in multicenter clinical trials. A quantitative alternative is the use of the MT Z‐spectrum and its associated pool size ratio (PSR), which is the ratio of the amount of MT in water vs. non‐water and has been significantly associated with myelin density as validated with histology.

CEST imaging takes complementary advantage of the saturation transfer effect between protons belonging to different chemical groups and bulk water.^39^ This approach allows for visualization of low concentration, exogenous or endogenous chemicals and includes a battery of broadly related techniques named for the specific exchangeable protons targeted. For example, amide proton transfer (APT) techniques detect amides from mobile proteins and peptides^40^; AcidoCEST detects iopamidol or iopromide^41^ and GlucoCEsT detects hydroxyls from glucose.^42^

Compared to diffusion MRS, the MT and CEST methods can evaluate some of the same molecules that change concentration during neuroinflammation, such as Ins (due to its six exchangeable —OH groups in the molecule) and creatine (due to its —NH_2_ group). In vivo maps of Ins using CEST at 0.6 ppm have been created to study the inflammatory response to LPS injection in mouse models and human brain maps of Ins at 7 Tesla.^43^, ^44^ The MT based methods can also be designed to target different, larger molecules such as sugars (glycoproteins) and amides, which indirectly reveals myelin pathology and remyelination in animal models of multiple sclerosis and patient studies related to neuroinflammation.^45^, ^46^ The CFM pointed to limitations of CEST and MT, including sensitivity to temperature and pH environment as well as to radiofrequency and main magnetic field inhomogeneities that are unrelated to inflammation. To achieve improved repeatable and more accurate quantification in CEST and MT, some groups are exploring fingerprinting‐based reconstruction and artificial intelligence frameworks to uncover rapid acquisition protocols for parameter quantification.^47^ These technical advances may enable multi‐pool fitting with higher SNR and real‐time analysis even on 3 T human scanners that may support dissemination of the new techniques for broader use in clinical and research studies on neuroinflammation.

## Newer Avenues for Imaging Neuroinflammation

A critical initial decision entails separating peripheral from local (i.e., CNS) inflammatory processes. While the distinction can be challenging, newer techniques have been proposed to identify the presence of individual cell types and mediators through basic scientific imaging studies. The possibility of probing for macrophage/monocyte, lymphocytic, neutrophilic, plasma cell, eosinophilic, and mast cell activity has been complemented by parallel strategies to identify specific mediators of neuroinflammation such as cytokines, chemokines, proteases, and reactive oxygen species, any combination of which could help inform unique signatures of disease. Importantly, there remains an ongoing need to improve discrimination of pathological inflammation from reparative or beneficial physiologic manifestations and “euflammatory” processes.^3^ Endothelial cell activation underpinning many inflammatory processes can be examined using antibodies bound to USPIO as discussed previously, potentially imparting exquisitely high specificity for surface molecular and immune cell epitopes, and myeloperoxidase‐activated exogenous contrast has been explored for its ability to detect myeloid and glial activation. Despite the extensive list of possible “liquid biomarkers,” for all such cases there remains the challenge of linking the presence of such biomarkers with specific brain locations or severity of disease. Noninvasive imaging biomarkers promise to advance the management of such cases with the potential for high spatial resolution and high lesion‐to‐tissue contrast.

Owing to a general lack of specificity for inflammatory disorders, most MRI techniques that assess neuroinflammation remain relatively limited as diagnostic or outcome prediction tools or to monitor therapeutic strategies when used alone. An era of precision and personalized health and medicine has motivated the development of new, multi‐modal approaches and high‐performance biomarkers in neuroimaging to measure neuroinflammation. More recently, the emergence of a growing number of PET‐MRI systems developed by multiple vendors has vastly expanded options for specialized co‐modal molecular imaging that leverages the relative benefits of both MRI and PET scanning in a single session. A powerful motivating example is found in techniques that image reactive astrocytes,^48^ including probes of acetate metabolism (such as various ^11^C and ^18^F molecular PET probes) or more commonly through monoamine oxidase‐B (for instance using (11)C‐(R)‐[1‐(2‐chlorophenyl)‐N‐methyl‐N‐(1‐methyl‐propyl)‐3‐isoquinolinecarboxamide]), TSPO, or I2‐imidazoline binding site. More generally, the advent of PET‐MRI systems allows the powerful integration of both modalities, leveraging PET's sensitivity (pico‐to nanomolar concentrations), and rapidly improving target specificity, with the spatial and contrast resolution and structural content afforded by MRI. A large and growing number of ^11^C‐and ^18^F‐bound radiotracers have been explored in inflammatory disorders spanning conventional diseases in the neuroinflammatory spectrum (eg, MS) to more nuanced neurodegenerative, cognitive, and psychiatric conditions including Parkinson's disease, Alzheimer's disease, amyotrophic lateral sclerosis, schizophrenia, and depression.^49^ Systems research with multi‐modal batteries of such techniques have succeeded in detecting specific cell types and defining surface markers of disease, holding the potential of individualized treatment for neurological disease during longitudinal monitoring of therapeutic response. We hasten to add that such PET‐MR systems may not at present be available in all methodologies, but their multimodal analysis can support interpretation of brain inflammation imaging.

## Examples of Clinical Applications of Neuroinflammation MRI Techniques

### Neuroinflammation in Autoimmune Diseases

Neuroinflammation can be triggered by exogenous or endogenous (i.e., auto‐immune) sources. Autoimmune neuroinflammatory disorders may be classified depending on their main effect in the brain. Of these, the demyelinating diseases and in particular MS, are the most prototypical.^50^ Although the clinical and radiologic appearance of these diseases differs between classes, the mechanisms of neuroinflammation and their effects are very similar to each other and to other causes of neuroinflammation and are briefly summarized here. Pathogens and external *noxa* trigger a series of reactions such as activation of resident immunocompetent cells (microglia, astrocytes), disruption of the BBB, and subsequently infiltration of peripheral immune cells, including macrophages and T cells. For instance, MS models have shown that in the early stages of this disease, peripheral T cells become activated, enter the bloodstream, migrate through the layers of the BBB and attack neurons, ultimately causing demyelination.^51^ The consequences of these mechanisms are manyfold and include demyelination, edema formation, GABA receptor upregulation, iron accumulation, and eventually apoptosis and cell loss/atrophy. Many of these mechanisms and consequences of neuroinflammation in autoimmune diseases align with pathophysiology that are detectable through the imaging contrasts described above.

The immunocompetent cells within the CNS, i.e., microglia and astrocytes, can be detected after becoming activated in response to peripheral immune cell infiltration. For instance, lactate and myoinositol increase after activation and can be measured with MRS, while receptors and molecules like Cox‐1, α4β2 receptor, and TSPO can be targeted with dedicated PET agents. TSPO in particular has gained attention, but has turned out not to be very specific.^6^ Second, disruption of the BBB can be readily visualized with gadolinium chelates since these molecules can cross the BBB in a similar manner as infiltrating peripheral immune cells. DCE‐MRI can be used for such purposes, as briefly discussed in the preceding paragraphs. There exist some PET agents that target BBB integrity, like ^11^C‐verapamil (targeting P‐glycoprotein), but MRI is the mainstay technique for assessing BBB integrity.^51^ The infiltration of immune cells, on the other hand, is less easily probed. Several PET and single‐photon emission computerized tomography (SPECT) agents have been used for this purpose (eg, ^18^F‐FDG, ^111^In, and ^99m^Tc), but these remain nonspecific, targeting the associated increased metabolism rather than cellular infiltration itself. MRI‐focused animal studies have been very promising, wherein immune cells are infiltrated with iron oxide particles and injected into the animals to visualize cellular infiltration and even single‐cell tracking^52^, ^53^; however, these techniques are not feasible (yet) in human studies.

Beyond the imaging of processes underlying neuroinflammation, attention has also been directed to visualizing its consequences. Loss of myelin and myelin repair are common in all autoimmune diseases in the brain. Demyelination can be targeted with PET using ^11^C‐PiB, whose binding is closely correlated to myelination, albeit with still indeterminate mechanisms. Interestingly, an *increased* MT effect was found in the CSF of patients with MS, even though CSF is thought to have little to no MT effect owing to its low macromolecular pool. This increased MT effect in CSF correlated with a *decreased* MT effect in spinal cord tissue in MS patients, which raises the question whether this phenomenon reflects myelin byproducts, end products, or part of the waste clearance system.^54^ To image apoptosis that may occur downstream of neuroinflammation, currently only PET and SPECT agents are available (^18^F‐ICMT‐II, ^99m^Tc‐Annexin V), while neuronal loss has been imaged with PET (^11^C‐flumazenil) and MRI (MRS for N‐acetylaspartate detection, mc‐relaxometry, voxel‐based morphometry and cortical thickness).^6^ Using MT in MS, studies found PSR was to be associated with functional outcome in relapsing–remitting MS, and to correlate with cognition in gray matter.^55^, ^56^

Finally, two emerging techniques merit attention. ^18^F‐MMP is a PET agent targeting matrix metalloproteinases, an enzyme class found to be involved in the degradation of the BBB in MS. This PET agent has shown promising results in both mice and humans, where it binds within MS lesions but has no correlation with the contrast‐enhancing parts of these lesions, reflecting a different imaging mechanism (BBB leakage vs. active BBB degradation).^57^ Also in MS, choroid plexus imaging has gained recent attention with the observation that the volume of the choroid plexus is altered during the disease, correlates with symptoms and extent of disease, and exhibits changes at an earlier stage than MS is generally seen on MRI.^58^

### Neuroinflammation in Tumors

**FIGURE 4:**
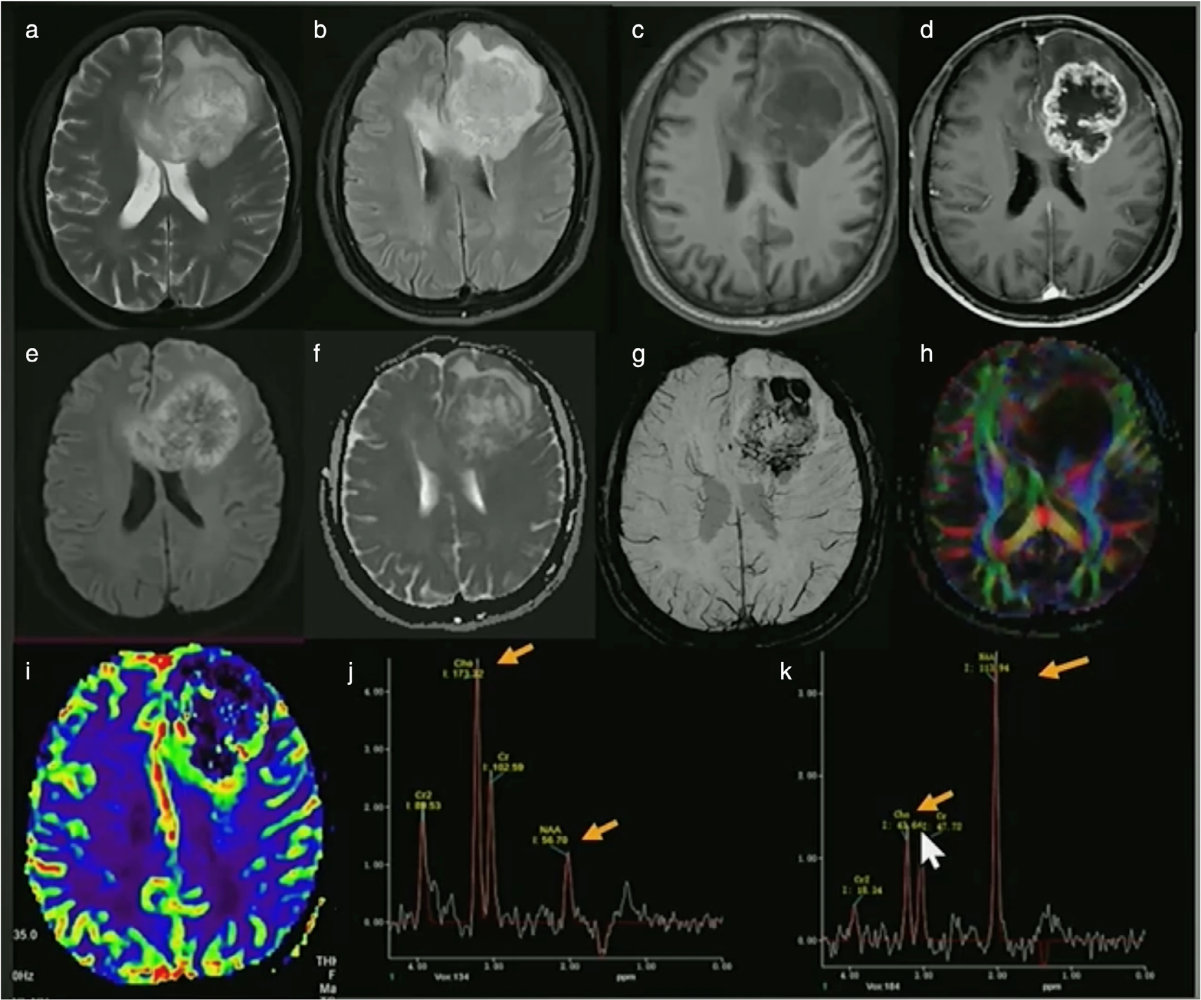
A 58‐year‐old male patient presented with headaches. Multimodal MRI showed a lesion in the left frontal lobe, extending to the right frontal lobe via the corpus callosum. The lesion was hyperintense on T2 (a) and FLAIR (b), hypointense on T1 (c), irregular rim enhancement on post‐contrast T1 (d), diffusion restriction on DWI (e) and ADC (f), multiple hypointensities on susceptibility weighted image (g), destruction of white matter tracts on diffusion tensor colored fractional anisotropy map (h), hyperperfusion on PWI (i), and markedly increased Cho and decreased NAA peak (j). In panel (k) a normal 1H‐MRS spectrum is shown. Pathology showed grade 4 glioblastoma. (By courtesy Qiang Yue, West China Hospital of Sichuan University, Chengdu, China.)

The inflammatory basis of neoplasia has evolved into a novel frontier of research, driven by the advent of immune‐modulating therapies. Multimodal MRI techniques are commonly employed in clinical practice to improve differential diagnosis, provide more tailored treatment, help minimize surgical risk, and facilitate monitoring of treatment response. For example, perfusion‐weighted imaging and DWl can help evaluate tumor vascularity and tissue cellularity and proton MRS can differentiate glioma from normal surrounding tissue (Fig. 4). Moreover, there is general opinion that the tumor microenvironment (TME) and its imaging in the context of neuroinflammation and associated pathophysiology can significantly improve understanding of the disease.^59^

TME refers to a complex evolving entity that contains tumor cells, blood vessels, and the extracellular matrix including fibroblasts and stromal cells within it. The TME is formed at an early stage of tumorigenesis, and there is a constant interaction between this environment and tumor cells, whereby each influences the other in both positive and negative ways. This interaction is facilitated by various mechanisms, including cell signaling pathways, recruitment of immune cells and components, the extracellular matrix, inflammation, tumor hypoxia, and even genetic reprogramming.^60^, ^61^ Low oxygen levels and an acidic environment facilitate tumor progression and migration.^62^ Additionally, the TME triggers the process of tumor angiogenesis. Four MRI techniques have shown promise in assessing the TME, including CEST, DTI, perfusion imaging and MRS combined with radiomic analysis.

**FIGURE 5:**
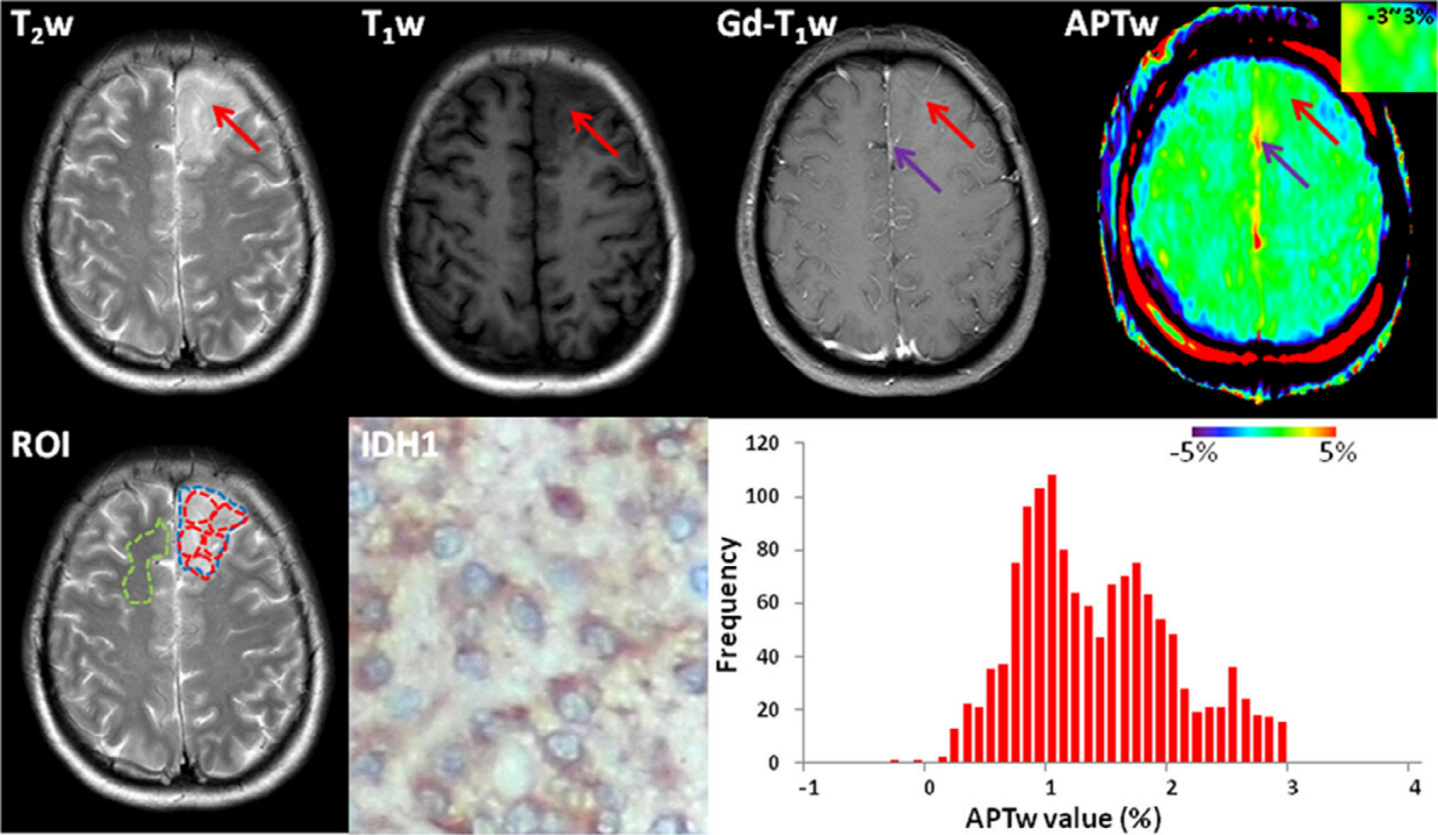
Histologically determined World Health Organization grade II oligodendroglioma IDH1 mutated tumor in a 52‐year‐old female. A T2‐weighted (T_2_w) hyperintense lesion with cortical thickening is seen in the left anterior frontal lobe, hypointense in T1‐weighted (T_1_w) and with no gadolinium enhancement (Gd‐T_1_w). The APTw (amide proton transfer weighted) image shows an ill‐defined iso to hyperintense lesion in the lesion. Purple arrows in Gd‐T1w and APTw images indicate a large vessel. The whole‐tumor APTw histogram had a mean APTw value of 1.37% and a 50th percentile APTw value of 1.30%. Purple arrows indicate a large vessel. Quantitative analysis and histogram plots were derived from ROIs for placed on the T2w image: Red dashed ROIs delineate five tumor ROIs, the blue dashed ROI for the whole tumor. The green dashed ROI indicates the normal white matter on the opposite frontal lobe.^64^

One of the most developed CEST based contrast is APT imaging, which has shown potential to differentiate high‐grade from low‐grade brain tumors. APT MRI signal contrast is determined by the exchange of amide protons and bulk water signal.^63^ In tumors, the local protein concentration usually increases due to cellular proliferation and accompanying neuroinflammatory processes creating with a more acidic environment that leads to an increase in APT‐weighted (APTw) signal. The latest 2021 WHO classification of CNS tumor relies heavily on isocitrate dehydrogenase (IDH) mutation status given its considerable diagnostic and predictive values: for example, the presence of IDH mutation in gliomas is linked to more favorable prognosis. APTw imaging is increasingly being used clinically to detect IDH mutations in gliomas, thereby improving patient management^64^ (Fig. 5). In recent years, APT derived radiomics has been used to predict IDH mutation status in gliomas. Radiomics collectively characterize and quantify pools of biological data and translate these into the structure, function, and dynamics of an organism.^65^ Other CEST based imaging used in tumors is AcidoCEST MRI, which can measure tumor extracellular pH thereby identifying the presence of acidosis in TME which is related to its metastatic potential.^66^

These technical advances in MRI have the potential to be applied clinically for investigating tumor potential for metastasis and to investigate potential therapeutic approaches that can mitigate neuroinflammation and improve the prognosis of tumor patients.

### Pediatric Neurological Disorders and Neuroinflammation

Throughout early development, both the anatomy and physiology of the brain undergo rapid changes.^67^ Variation in myelination levels, the ongoing development of synaptogenesis and functional and structural neural circuitry, increased cerebral metabolism and the degree of vascularity are all factors influencing the response of the developing brain to injury. The viscoelastic properties of gray and white matter are also different, making them more prone to certain diseases such as traumatic brain injury.^68^ Given the unique characteristics of the pediatric brain, prompt recognition of CNS inflammation is paramount to mitigating long‐term disability in brain development.

The most common neuroinflammatory condition in the pediatric population is monophasic acute disseminated encephalomyelitis (ADEM). This is in contrast with the more frequent polyphasic MS disease in adults. MS in the pediatric population is rare, affecting 2%–10% of all patients with MS.^69^ Together with the age at onset of disease, the risk of developing MS in pediatric patients with suspected acute demyelinating syndrome heavily relies on MRI features.^70^ The accurate diagnosis will also determine whether the disease will remain monophasic or will recur over the years allowing for novel targeted therapies. In the last decade, MRI‐defined phenotypes appear to be more sensitive in risk stratification of disease and in differential diagnosis of pediatric neuroinflammation which has implications for treatment strategies.^71^, ^72^

**FIGURE 6:**
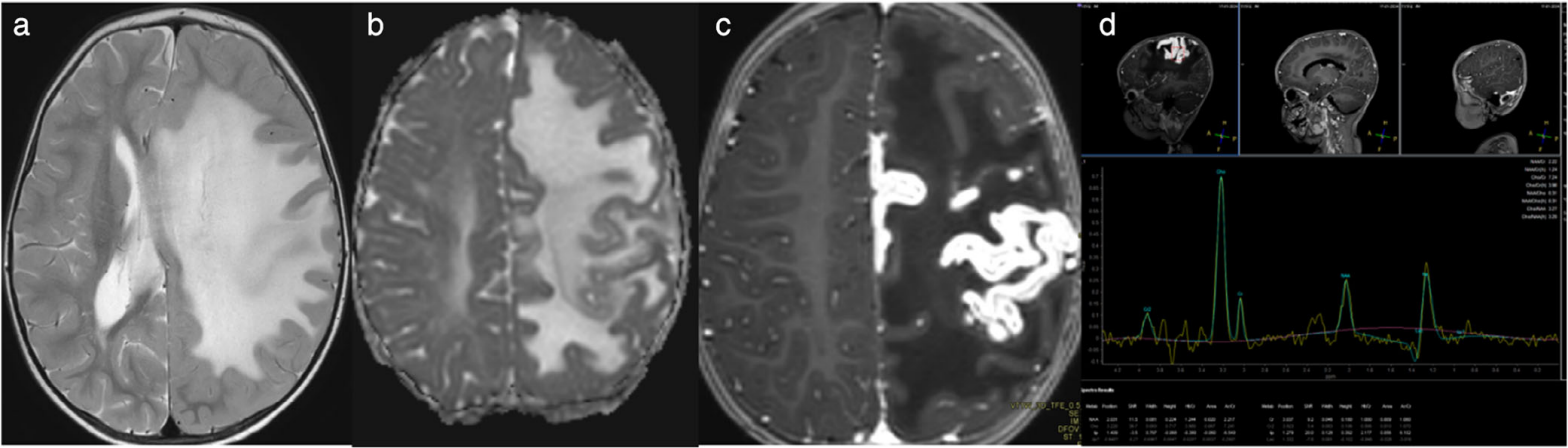
A 2‐year‐old boy with reactivation of Herpes simplex encephalitis. (**a**) T2w axial image shows a large white matter hyperintense area in the left hemisphere with mass effect on the lateral ventricle and a mid‐line shift. (**b**) Axial ADC map shows increased signal in the corresponding left hemisphere indicative of vasogenic edema. (**c**) Post gadolinium T1 image shows avid enhancement of several frontal gyri suggesting damage of the BBB. (**d**) Single voxel short TE 1H‐MRS shows very high choline, low NAA, and high levels of lipids.

Several immune‐mediated conditions, distinct from MS, often masquerade as epileptic seizures or neuropsychiatric disorders, potentially escaping diagnosis.^73^ Vaccinations, underlying cancers, infections, or, in many cases, an unidentified cause triggers antibody production. During the CFM, special emphasis was placed on the pivotal role of MRI in the differential diagnosis of disorders characterized by abnormal production of antibodies targeting brain neurotransmitter receptor antigens like NMDA receptor, GAD‐65, and myelin oligodendrocyte progenitor protein (MOG)^73^ (Fig. 6). The patterns observed on MRI scans reflect the distribution of CNS receptor density in the CNS. The severity of lesions will depend on the degree of brain development at the time of disease in terms of ongoing myelination or neural network development. There also exists possibility of disrupted connectivity which may lead to a range of disability and loss of cognitive skills. Widespread damage to white matter leads to reduced functional connectivity resulting in cognitive impairment.^74^

**FIGURE 7:**
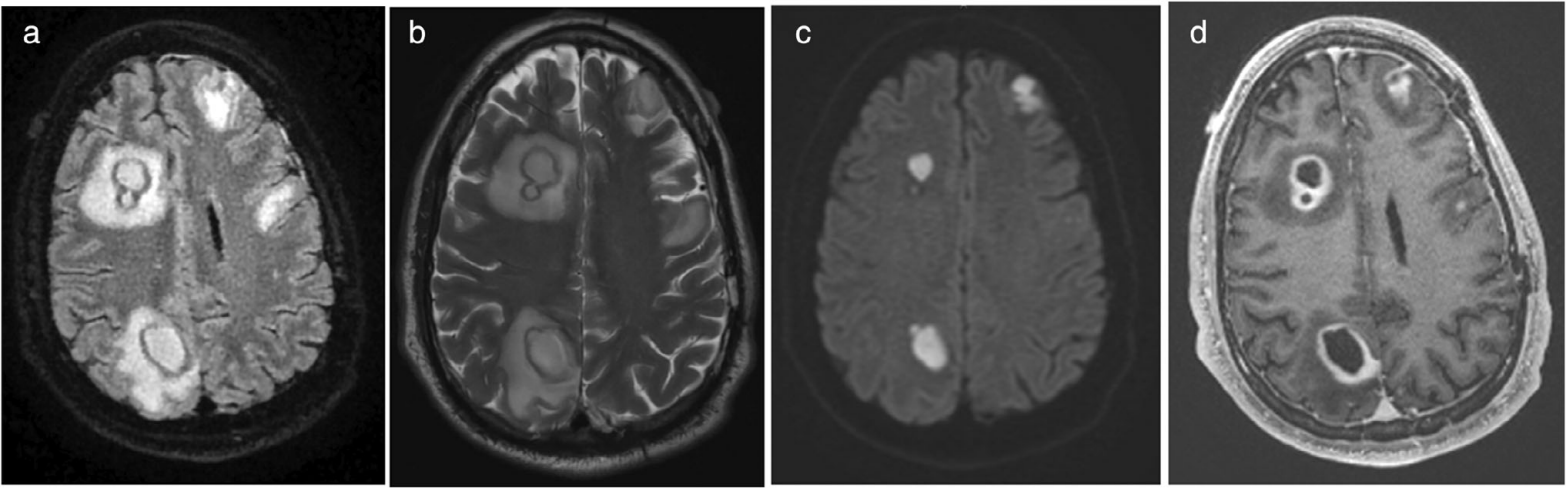
An example of bacterial infection: (**a**) Axial FLAIR image shows multiple cystic lesion characterized by a hypointense rim and surrounded by white matter hyperintensity. (**b**) Axial T2 weighted images shows signal characteristics similar to FLAIR. (**c**) Axial DWI based trace image shows restricted diffusion within the cystic lesions and (**d**) axial post gadolinium based T1 weighted image identifies thick rim surrounding cystic lesions which is significant for blood brain barrier disruption. (Courtesy John D. Port, Mayo Clinic, Rochester.)

A range of infectious agents, including viruses, bacteria, protozoa, and fungi, can trigger brain infections in pediatric patients (Fig. 7). The severity of these infections depends not only on the type of microorganism but also on the developmental stage of the fetal, neonatal, and infantile brain at the time of infection. These agents can provoke a spectrum of neuroinflammatory responses, including microglial activation, heightened permeability of the blood–brain barrier, and initiation of the immunological cascade within the parenchyma.^75^, ^76^ Pseudo‐continuous arterial spin labeling (pCASL) represents an effective and recent advancement facilitating the evaluation of cerebral blood flow and providing insights into BBB integrity. This gadolinium‐free, noninvasive technique has the potential to assess BBB integrity in the developing brain and is recommended for inclusion in the standard clinical MRI protocol for investigating brain infections.^77^ Nonetheless, gadolinium‐based imaging still remains crucial, especially in the initial evaluation of patients for detecting meningeal involvement, the presence of proteinaceous material in the CSF spaces, potential vasculitis, and more minute lesions. Post‐contrast FLAIR imaging demonstrates superior sensitivity in detecting meningeal involvement compared to T_1_‐weighted post‐contrast imaging.^78^

**FIGURE 8:**
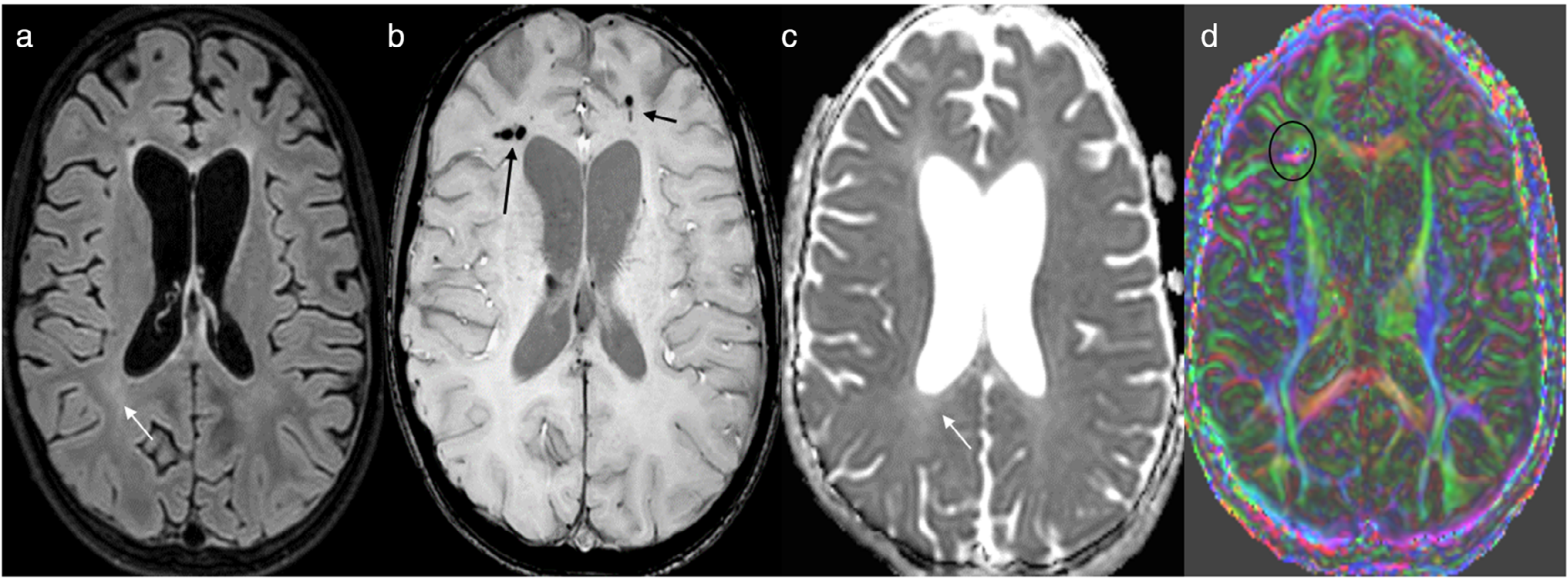
A case study of a 12‐year‐old male with traumatic brain injury: (**a**) An axial FLAIR image reveals subtle diffuse hyperintensity (white arrow) in the white matter and enlarged ventricles indicative of progressive atrophy. (**b**) An axial SWI demonstrates multiple hypointensities along the bilateral frontal white matter tracts, reflecting microbleeds (black arrows). (**c**) Axial ADC maps exhibit increased signal throughout the white matter, confirming elevated extracellular water content (white arrow). (**d**) A color‐coded FA DTI map highlights disrupted white matter and a thin corpus callosum genu. Additionally, hemosiderin in the right frontal lobe distorts the DTI signal (indicated by a black circle).

The annual incidence of traumatic brain injury (TBI) affects between 27 and 69 million individuals worldwide, imposing a significant socio‐economic burden.^79^ Primary brain injuries manifest as skull fractures, epidural or subdural hematomas, subarachnoid, and intracranial hemorrhages. Secondary consequences of TBI include increased excitotoxicity, mitochondrial/metabolic abnormalities, heightened oxidative stress, cytoskeletal degradation, impaired cerebral blood flow or dysregulation thereof, and increased edema in the interstitial space.^80^ Compared to the adult brain the developing pediatric brain is more susceptible to external forces due to ongoing myelination, synaptogenesis, weaker neck muscles, and a less rigid skull.^80^ Sutures may reopen without producing clinical symptoms until late stages. The extent of the neuroinflammatory response depends on magnitude of force and the resultant damage.^81^ Recent evidence suggests that TBI can increase the deposition of tau and amyloid proteins, leading to cognitive dysfunction known as chronic traumatic encephalopathy, which is a significant area of research in TBI.^82^ Imaging plays a valuable role in accurately visualizing microstructural damage to white matter and identifying petechial hemorrhages, also known as diffuse axonal damage (Fig. 8). ASL‐based perfusion imaging provides insights into neurovascular changes resulting from trauma.^77^, ^83^ DWI and DTI are valuable techniques to estimate the extent of microstructural damage in the acute and chronic phases of neuroinflammation. Diffusion kurtosis imaging, neurite orientation dispersion and density imaging have successfully measured changes in white matter abnormalities and links to specific cognitive domain dysfunction in patients with TBI.^84^

Research in the pediatric population presents numerous challenges. There is a need for improved MRI methods are needed to identify neuroinflammatory changes in pediatric brains in the backdrop of physiological changes during brain development. This necessitates higher resolution and less invasive MRI techniques that are easily repeatable over time. To reduce the need for sedation in very young children, appropriate hardware together with faster sequences would be needed.

## The Role of the Brain‐Gut Axis in Neuroinflammation

Neuroinflammation is often delimited as a process developing strictly within the brain, with little connection to the rest of the body apart from potential pathogens arising from outside. However, in recent years new insights have pointed to compelling links between a so‐called brain‐gut axis. The brain‐gut axis describes a complex and bidirectional interaction between microbiota within the gut and the brain. Peripheral effects of the microbiota that have been recognized are immune system dysregulation, peripheral inflammation and a leaky gut, while central effects are thought to include neuroinflammation, BBB disruption, and amyloid aggregation, the latter of which is important in Alzheimer's disease (AD) pathogenesis.^85^ Indeed, the brain‐gut axis has gained attention in light of its role in dementia, and in particular AD. Dementia is a growing global health concern and an umbrella term for varying underlying diseases, the most common of which is AD. Apart from Aß plaques and tau protein, the pathogenesis of AD remains unclear with other factors like neuroinflammation and microvascular dysregulation as potential modulators of the disease process. It is currently thought that the gut microbiota can regulate neuroinflammation by producing byproducts and activating peripheral immune cells, leading to microglia activation and astrocyte stimulation, ultimately upregulating the latter into reactive astrocytes that cause damage to neurons and promote the development of Aß plaques.^86^ Indeed, studies have shown that AD is associated with characteristic gut metabolomic signatures.^85^

**FIGURE 9:**
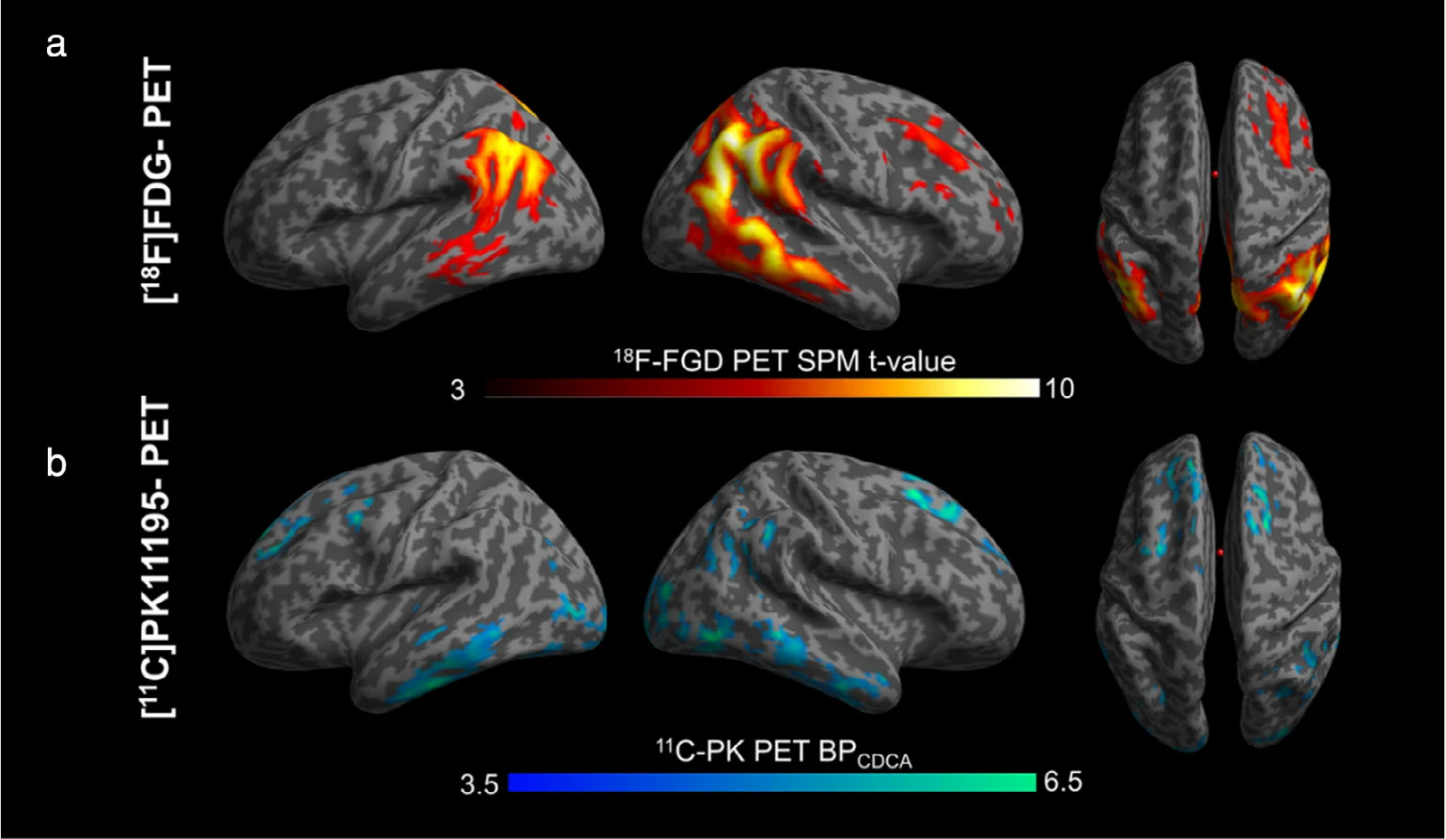
This figure illustrates differences in glucose metabolism and microglial activation between ì patients with early onset Alzheimer's disease and healthy controls using two PET imaging agents: ^18^F‐FDG, which is specific to glucose metabolism, and ^11^C PK 11195, which targets microglial activation. (**a**) Areas of the brain showing areas of hypometabolism and (**b**) areas showing binding to ^11^C PK 11195 in areas of increased microglial activation, a surrogate of neuroinflammation. Color bars represent ^18^F‐FDG and ^11^C‐PK 11195 levels of significance.^87^

Imaging of the brain‐gut axis is currently limited to visualizing the associated pathways leading to neuroinflammation, and has focused primarily on AD‐associated neuroinflammatory biomarkers with PET. PET agents that have been used for this purpose include agents that target enzymes or signaling molecules (eg, ^11^C‐PK11195, ^11^C‐DAA1106, ^11^C‐PBR28), and agents that target G‐protein coupled receptors (eg, ^11^C‐NE40, ^11^C‐SMW139, ^11^C‐P2Y12R). Some of these agents have already been applied in human clinical studies, with promising results^87^ (Fig. 9). However, specific MRI techniques to address the brain‐gut axis are relatively sparse, and much work is still needed to elucidate this integral and intricate communication pathway.^88^

## Neuroinflammation and Brain Waste Clearance Pathways

**FIGURE 10:**
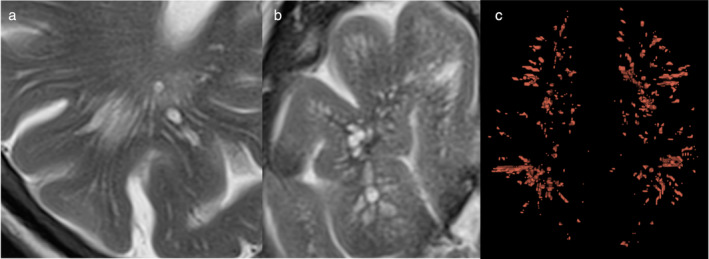
Enlarged perivascular spaces (ePVS) in the human brain. (**a**) Axial T2 weighted image shows multiple elongated ePVS in the deep white matter of the right parietal lobe. (**b**) Axial T2 weighted images showing multiple ePVS in the white matter of the right temporal lobe with cyst‐like morphological changes. (**c**) Automatic segmentation using deep learning algorithm of ePVS in a subject with autism spectrum disorder.

Recently described brain waste clearance pathways such as the glymphatic system and the intramural periarterial drainage pathways facilitate the drainage of parenchymal interstitial fluid (ISF) toward the subarachnoid space and the cervical lymph nodes.^89^, ^90^ The ISF contains macromolecules and waste products such as Aß and tau which may deposit and trigger neuroinflammation. Perivascular spaces (PVS) are a common denominator in all clearance pathways making them a proxy for altered drainage of ISF and macromolecules in neurological diseases.^91^ Indeed, these spaces enlarge if drainage of ISF is blocked due to accumulation of macromolecules^92^ (Fig. 10). Visual rating scales have been employed by neuroradiologists to determine the count and the distribution of PVS in the centrum semiovale and the basal ganglia, however, this can underestimate findings.^93^ An automated segmentation approach would allow for more accurate characterization of PVS. One such method is increasing the signal contrast of PVS by creating enhanced perivascular contrast derived from T1 and T2 weighted images^94^ (Fig. 10). Such a segmentation approach has been used to correlate PVS volume fraction and neuroinflammatory serum markers successfully in the basal ganglia, although not so in the centrum semiovale.^95^ PVS are naturally present in healthy individuals and are correlated with age, gender, body mass index and the scan time during the day.^96^, ^97^ DTI has been applied to identify fluid motion within the PVS by assuming that fluid will be restricted in case of blocked drainage with respect to free flowing ISF filled PVS.^98^ Low FA and high MD are present in areas with larger PVS volume fraction. In two compartment DTI models, the non‐parenchymal fluid appears to contribute more to mean diffusivity changes in pathology such as mild cognitive impairment with respect to patients with normal cognition.^99^ Taken together, MRI can be used to measure PVS volume and evaluate its morphology by improving segmentation methods that incorporate multiple MRI contrasts. Multi‐shell DTI two‐compartment models may allow for better delineation of diffusion properties of fluid within the PVS.

## Clinical Translation—Are We There Yet?

The preceding summarizes show increased insight into neuroinflammatory mechanisms combined with new MRI techniques may advance our knowledge of the role of inflammation in neurological diseases (Table 1). However, using this knowledge to diagnose, treat, and cure individual patients requires clinically applicable interventions that help facilitate clinical workflow. The central vein in MS lesions is a good example of clinical translation of MRI techniques because of its relative ease of identification on routine T_2_*‐weighted image and is used to differentiate MS lesions from white matter lesions due to other causes. Great strides have also been made to identify subtle BBB leakage in neurodegenerative diseases, deposition of iron in neurodegenerative diseases, and to evaluate metabolic changes in acute and chronic brain diseases. Advancements in MRI hardware and sequence development are geared toward faster acquisition without compromising resolution. While vasculitis has not specifically been discussed in detail during the CFM, intracranial vessel wall MRI has been used in many clinical centers for several years to identify inflammation of the vessel wall.^100^ From this CFM, it is evident that not all technical advancements and improvements are being translated into patient care. Achieving this demands collaboration between clinicians and scientists to prioritize pertinent research inquiries. Moreover, it necessitates active involvement from stakeholders, including patients, caregivers, funders, and industry players, at every stage of clinical research. Engaging patients and caregivers throughout their clinical care is necessary to foster mutual trust, leading to enhanced patient recruitment and retention and supporting robust clinical research programs.^101^, ^102^ Many funding organizations and clinical journals have advocated for researchers to conduct patient‐oriented research, with greater patient and public involvement in order to formulate research questions, develop suitable research protocols, interpret findings, and disseminate evidence effectively. Exposing healthcare workers, caregivers, and scientists to the clinical environment to this shared space is anticipated to represent a pervasive bottleneck undermining clinical advancement.

**TABLE 1:** Specific MRI Sequences That Can Be Used to Identify Neuroinflammatory Processes in the Brain

Technique	Advantages	Limitations	Acquisition	Postprocessing
FLAIR	Clinically available Detects edema Post gadolinium FLAIR is highly sensitive to cerebrospinal fluid enhancement	Non‐quantitative	Scans can achieve 1 mm isotropic resolution Select inversion time appropriate for T_1_ of water and field strength	Identify presence and measure edema volume Segmentation of FLAIR lesions through manual or histogram‐based delineation
Dynamic contrast enhanced MRI	Directly assesses blood–brain barrier permeability Quantification of kinetic parameter (K_trans_) is possible	Requires Gd contrast agent administration	At minimum, a T_1_‐weighted scan before and after contrast administration T_1_ mapping sequence can improve quantification Kinetic modeling to measure K_trans_ uses dynamic scans with 1–2 second temporal resolution or faster	Quantification of post—pre contrast T_1_‐weighted signal in key brain regions Kinetic modeling using Tofts model with input function derived from large artery signal time course in the image
T_2_*‐weighted and quantitative susceptibility mapping	Can be combined with new contrast agents (eg, USPIOs) that are taken up by inflammatory cells Sensitive to endogenous iron in glial cells without contrast agent	Multiple sources of iron signal and diamagnetic contributions may confound signal Limited specificity	Multi‐echo gradient echo acquisition is recommended for T_2_* mapping from magnitude signal Isotropic spatial resolution is preferrable for quantitative susceptibility mapping	T_2_* relaxation quantification from exponential decay of magnitude signal Quantitative susceptibility mapping from phase signal, with background field removal and dipole inversion (reconstruction)
Diffusion tensor imaging	Assesses white matter microstructural properties such as fractional anisotropy (FA) Free water (FW) values provide sensitive physiological characterization Differentiates cytotoxic from vasogenic edema	Limited specificity to neuroinflammation	For single‐shell DTI, sufficient orientation vectors (eg, 40) required for FA and FW measurement Multiple diffusion *b*‐values needed for advanced measures such as diffusion kurtosis	Distortion correction performed using fast, reversed phase‐encode polarity scan Diffusion tensor model with FW compartment Non‐Gaussian model enables measures of kurtosis
Spectroscopy	Detects specific metabolites, eg, choline, lactate, or myoinositol, with altered concentrations in brain inflammation	Limited signal‐to‐noise ratio Larger imaging voxels lead to limited spatial coverage	Single voxel scans volumes are a few cm^3^ Careful main field shimming in target region for imaging is needed Advanced spatial‐spectral encoding allows multiple slices in spectroscopic imaging	Linear combination model fitting of non‐apodized spectra to quantify metabolite peaks and ratios Cellular compartment analysis for combined diffusion‐weighted spectroscopy
Magnetization transfer (MT)/Chemical exchange saturation transfer (CEST)	Uses off‐resonance saturation pulses to target signal from specific inflammation‐related metabolites and larger molecules and proteins Improved signal‐to‐noise compared to spectroscopy	Sensitive to non‐specific effects of temperature and pH Typically semi‐quantitative	Careful B_0_ field shimming, preferably second order, is needed Continuous‐wave or pulse train saturation with optimized parameters for target metabolite Water frequency mapping is desired	Quantification of asymmetry ratios such as MTR at specific frequencies for target metabolites Advanced workflows perform spectral modeling of the Z‐spectra lineshape in CEST

An ad‐hoc live poll during the follow‐up CFM session at the 2024 ISMRM Annual Meeting, where researchers could show their latest advancements in neuroinflammation imaging, showed that many researchers and clinicians alike have started to use neuroinflammation sensitive MRI sequences clinical care in hospital settings.
